# Screening Heterofermentative Lactic Acid Bacteria as Silage Inoculants for Osmotolerance †

**DOI:** 10.3390/microorganisms14010117

**Published:** 2026-01-05

**Authors:** Siriwan D. Martens, Wolfgang Wagner, Mariana Schneider, Klaus Hünting, Susanne Ohl, Christof Löffler

**Affiliations:** 1 Saxon State Office for Environment, Agriculture and Geology (LfULG), 04886 Köllitsch, Germany; 2 Center for Agricultural Technology Augustenberg (LTZ), 76227 Karlsruhe, Germany; 3 Bavarian State Research Center for Agriculture (LfL), 85586 Poing, Germany; 4 Chamber of Agriculture North Rhine-Westphalia, 47533 Kleve, Germany; 5 Chamber of Agriculture Schleswig-Holstein, 24327 Blekendorf, Germany; 6 Agricultural Centre for Cattle Production, Grassland Management, Dairy Food, Wildlife and Fisheries Baden-Württemberg (LAZBW), 88326 Aulendorf, Germany

**Keywords:** growth medium, high dry matter, in vitro, protocol, wilting

## Abstract

Heterofermentative lactic acid bacteria (LABhe) can help to increase aerobic stability of silages. As high dry matter silages are especially prone to aerobic deterioration, the question was whether an in vitro test could help to select for LABhe with tolerance to these conditions, that is, to high osmolality. A medium proven for homofermentative lactic acid bacteria (LABho) with high sugar concentration was applied in vitro while validating the results in high DM grass silages in situ. The pH was measured at 0, 24, 36 and 48 h or after 5 d as indicator for the bacterial fermentation activity and compared. As a result, LABhe showed to be far less susceptible to high DM and osmolality respectively, compared to LABho. It was concluded that the in vitro test allows for qualitative assessment of the osmotolerance of LABhe, but not for quantitative ranking between LABhe strains.

## 1. Introduction

New silage inoculants are constantly being developed to be more forage specific, adapted to specific climate conditions and dry matter (DM) levels. Simple laboratory protocols are needed to speed up the selection process while reflecting field conditions. The evaluation of a protocol presented in this article (expanded version of conference abstract [1]) makes a contribution to this task. One of the biggest challenges of silage-making is the aerobic instability in the feedout phase. Although increasing forage dry matter by wilting is a recommended good agricultural practice [2] to enhance nutrient concentration, limit proteolysis and inhibit silage effluent, it makes compaction more difficult and results in increased pore volume in the silo stock and thus risks air ingress and aerobic deterioration. On the other hand, it may impact the viability of lactic acid bacteria (LAB) in the initial fermentation phase [3]. Obligate heterofermentative lactic acid bacteria (LABhe) are able to form acetic acid from water soluble carbohydrates, among others, which possess fungistatic characteristics and thus decelerate aerobic spoilage. Thus, they are recommended inoculants for this purpose. The question was whether it was possible to select for LABhe specifically adapted to and suitable for high dry matter conditions with a newly developed protocol [4]. Drought stress corresponds to high osmotic pressure [5]. In a culture medium it can be varied by adjusting the osmolality, i.e., the concentration of osmotically active particles (solutes) in a liquid solution. Care has to be taken when using salts as not all LAB strains exhibit halotolerance [6] while still tolerating certain levels of drought. The aim of the study was to examine whether the newly developed culture medium [4] was equally suitable for LABhe strains as was shown for homofermentative ones (LABho), and how LABhe behaved in situ under high forage DM conditions (≥40% DM) compared to average DM conditions. The indicator for LAB activity, and thus viability, was the pH decrease both in vitro and in situ [7,8].

## 2. Materials and Methods

The experimental setup included an in vitro part with culture media representing conditions of low and high osmotic pressure and an in situ part ensiling forages at different dry matter levels to validate the results obtained in vitro with the same LABhe strains as inoculants.

### 2.1. In Vitro Trial

The reference culture medium for low osmolality (0.4 osmol/kg) was MRS broth (MRS Broth pH5.7, Art. No. HP64.1, Carl Roth GmbH+ Co. KG, Karlsruhe, Germany). High DM grass (~45% DM) was simulated with modified “High Sugar” (HS) MRS broth with an osmolality of 2.4 osmol/kg [5]: MRS as dehydrated culture media (54 g) plus 100 g glucose and 100 g fructose, along with 28 g KCl dissolved in 1.0 L distilled water, and adjusted to pH 5.7 using NaOH (1 M) [4]. Aliquots of 10 mL in test tubes were heated to 100 °C for 2 min for sterilization. The modification compared to the published medium was in aligning the pH of the HS MRS to the standard MRS while maintaining the target osmolality, and reducing the heat and time for sterilization to not change the pH again while still achieving sterile conditions.

Biological silage additives were inoculated according to the manufacturer’s recommended dosage, at which 1 g of forage is equivalent to 1 mL of culture medium.

Each product was inoculated in triplicate in both the standard and the HS MRS broth. Uninoculated media served as reference.

The pH of the uninoculated media was measured (0 h) and the treatments after 24, 36 and 48 h of incubation at 30 °C, disinfecting the pH electrode between treatments.

### 2.2. LAB Inoculants

Five different commercially available products were tested, four of them containing different strains of the obligate heterofermentative *Lentilactobacillus buchneri* exclusively (LAB1-4) (DSM 13573, DSM 22501, CNCM I-4323, ATCC PTA-2494), and one containing a mixture of *L. buchneri, L. diolivorans* and the facultative heterofermentative (commonly referred to as homofermentative) *Lacticaseibacillus rhamnosus* (LAB5) (SBE 10070, NCIMB 30141, LAC 1129). All of the freeze-dried products were dissolved in sterile Ringer solution in a defined concentration before inoculation in the culture media.

### 2.3. In Situ Trial

In May–July 2024, grasses from pasture at five different locations in Germany (South, Southwest, West, North and East Germany) were cut and wilted to achieve a target DM of 35–49%. They were chopped, and six treatments applied in at least triplicate: an uninoculated control plus the five inoculants LAB1–LAB5. The forages were ensiled either in small WECK^®^ jars (Weck glass and packaging GmbH, Bonn, Germany) or vacuum sealer bags (≤1.0 L volume) for 5 days, stored at room temperature. A time of 5 days of anaerobic storage was chosen in contrast to 3 days with LABho as LABhe growth is slower, and acetic acid a weaker acid than lactic acid.

The DM content (drying at 105 °C for 24 h) and pH were determined before ensiling and pH after ensiling using a pH meter with a freshly calibrated electrode.

### 2.4. Statistical Analysis

A linear regression analysis between DM content and final pH value of inoculated grass silages was performed using SigmaPlot (V12, Systat Software Inc., San Jose, CA, USA). The final pH values of in vitro treatments in standard and HS MRS were compared by variance analysis using SPSS (V19, IBM SPSS Company, Armonk, NY, USA) considering the effects of media and inoculants.

Y_ij_ = µ + MED_i_ + INOC_j_ + ε_ij_ (1)

where µ = general mean, i = 1, 2 (medium with low/high osmolality), j = 1, 2, …, 5 (inoculant LAB1-5), and ε_ij_ = residual error.

Likewise, the silages were grouped in two or three DM levels and the final pH values of in situ treatments were compared considering the effects of DM level and inoculants.

Y_ij_ = µ + DM_i_ + INOC_j_ + ε_ij_ (2)

where µ = general mean, i = 1, 2, 3 (DM level [35 and 45 or 30, 40, 50%]), j = 1, 2, …, 5 (inoculant LAB1–5), and ε_ij_ = residual error.

## 3. Results

### 3.1. In Situ Results

**Figure 1:**
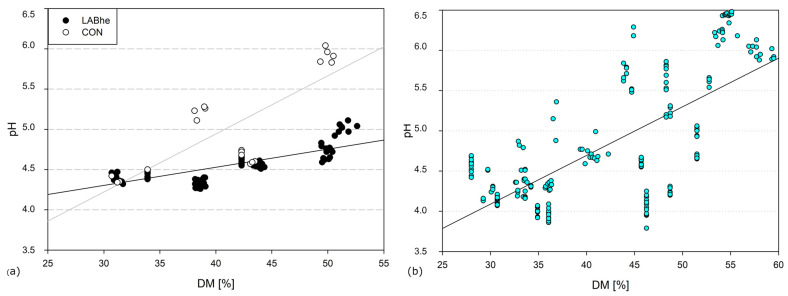
Linear regression between DM content and pH value of grass silages inoculated with (**a**) predominantly heterofermentative lactic acid bacteria (LABhe) or uninoculated CONtrol, (**b**) homofermentative LAB [4].

The actual achieved DM ranged from 30.7–52.6% (median 38.8%) in the grass silages. In four out of five locations, CON behaved similarly to the inoculated grass regarding the pH decrease during the first 5 days of ensiling, in relation to the actual DM content. In one location, the pH of CON remained higher (5.61 vs. 4.58 on average, Figure 1a).

There was an effect of DM level (*p* < 0.001) and inoculant (*p* < 0.01) in the two level’s DM grouping (<40 and ≥40% DM, alias 35 and 45, Table 1a), while there was no interaction between DM level and inoculant (*p* > 0.1). Only when divided in three groups (30–35, >35–45, >45% DM, alias 30, 40, 50), there was interaction between DM and inoculant (*p* < 0.01) (Table 1b). Higher DM resulted in higher pH (Table 1). Lowest pH was achieved with the mixed product LAB5 (Table 1).

There was a significant correlation between DM content and pH, while the gradient was shallow for the inoculated treatments (r^2^ = 0.60, *p* < 0.001, m = 0.02; Figure 1a).

### 3.2. In Vitro Results

**Figure 2:**
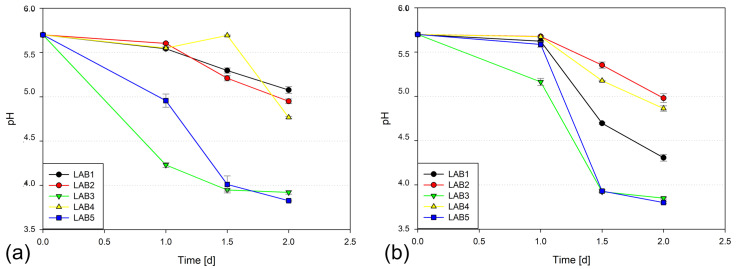
pH development in (**a**) standard MRS, (**b**) HS MRS during 48 h of incubation.

The pH decline in the two media following inoculation is presented in Figure 2.

Regarding each point in time separately (24, 36, 48 h), there was a significant interaction of inoculant and medium (*p* < 0.001). The average pH after 24 h of incubation of the 5 inoculants was significantly higher in HS MRS than in MRS: 5.55 (SEM 0.008) vs. 5.18 (SEM 0.009). After 36 h, this general difference slightly reversed: 4.62 vs. 4.83 (SEM 0.009), and after 48 h, 4.36 vs. 4.51 (Table 2).

The overall ranking of LAB products was similar for LAB2, 3 and 5 compared to the in situ results.

## 4. Discussion

The in situ results hint at the phenomenon that osmotic stress affects the fermentation capacity of LABhe to a lesser degree than LABho. The linear regression curve relating DM to pH of LABhe was much flatter than that of LABho (Figure 1). In a study of Driehuis et al. [9], *L. buchneri* fermented well when comparing pH of grasses of around 30% DM to grasses up to 42% DM. The same applied to maize when pH and fermentation products at 33% vs. 41% DM were compared [10]. *L. buchneri* seems to be rather unsuitable for low-DM conditions (<25% DM), leading to high losses and ammonia formation, and fermentation shifting to acetic acid and ethanol formation rather than lactic acid [11].

The tested culture medium impeded the metabolic activity of LABhe in the initial phase (24 h) compared to the standard medium, showing an effect of increased osmolality. However, the inoculants overcame this obstacle more or less quickly. It seems that in the HS MRS, LAB1 achieved the lower final pH by metabolizing the surplus of fructose and glucose to additional lactic and acetic acid compared to the standard MRS. On the other hand, pH of LAB2 and 4 remained > 4.5 after 48 h of incubation, which rather corresponded to the mean pH value achieved with LAB1-5 at 50% DM in situ. In another in vitro trial ending only after 72 h of incubation (not presented here), the pH of those LAB further dropped pointing to slower metabolism and/or growth. As a secondary response to osmotic stress, LAB may take up so called osmoprotectants such as glycine betaine, glycine, choline, proline, carnitine [12]. MRS contains all of these as components of yeast and beef extract [13], which may have helped in the end.

A clear statistical grouping of pH results of low DM and standard medium versus high DM and HS medium as achieved with LABho [4] was not possible here. As a consequence, the question arises as to whether the in vitro approach is suitable for osmotolerant LABhe or not.

There are two aspects to this question:

(1) The tested inoculants apparently are not as sensitive to high DM (high osmolality) as LABho as shown in situ and that might be generalized to *L. buchneri* strains.
(2) The in vitro test may still serve as control of osmotolerance rather than a selection tool for LABhe.

That means that from the in vitro approach, only a qualitative statement can be concluded for LABhe: if the pH after 48 h of incubation has dropped significantly (*p* < 0.05) compared to 0 h, then the strain/product is sufficiently osmotolerant. No ranking between strains/products can be derived.

## Figures and Tables

**Table 1:** Final pH of inoculated grass silages divided in (a) two DM levels (<40%, alias 35 and ≥40 DM %, alias 45), (b) three DM levels (30–35, >35–45, >45% DM, alias 30, 40, 50) after 5 d of ensiling.

**(a)**									
**DM level**	***n***		**LAB1**	**LAB2**	**LAB3**	**LAB4**	**LAB5**	**Mean**	**SEM**
35	50		4.42	4.41	4.40	4.42	4.34	4.40 ^B^	0.016
45	50		4.76	4.78	4.65	4.64	4.59	4.68 **^A^**	0.016
Mean			4.59 ^a^	4.59 ^a^	4.52 ^ab^	4.53 ^ab^	4.46 ^b^	4.54	0.012
**(b)**									
**DM level**	***n***	CON *	**LAB1**	**LAB2**	**LAB3**	**LAB4**	**LAB5**	**Mean**	**SEM**
30	30	4.44	4.47	4.44	4.47	4.44	4.37	4.44 ^B^	0.019
40	50	4.88	4.50	4.51	4.47	4.51	4.45	4.49 ^B^	0.015
50	20	5.92	5.00	5.04	4.75	4.72	4.65	4.83 ^A^	0.023
Mean		5.00	4.59 ^a^	4.59 ^a^	4.52 ^ab^	4.53 ^ab^	4.46 ^b^		

* For reference, not included in the statistical evaluation. Different superscript letters in upper case ^[A,B]^ indicate significant differences between DM level, in lower case ^[a,b]^ between LAB products (*p* < 0.05).

**Table 2:** Final pH of culture media (standard or High Sugar MRS) inoculated with LABhe for 48 h.

DM Level	*n*	LAB1	LAB2	LAB3	LAB4	LAB5	Mean	SEM
Stand. MRS	15	5.08	4.95	3.92	4.77	3.83	4.51 ^A^	0.007
HS MRS	15	4.31	4.98	3.85	4.86	3.80	4.36 ^B^	0.007
Mean		4.69 ^c^	4.97 ^a^	3.89 ^d^	4.82 ^b^	3.81 ^e^	4.43	0.005

Different superscript letters in upper case ^[A,B]^ indicate significant differences between the media, in lower case ^[a–e]^ between LAB products (*p* < 0.05).

## Data Availability

The original contributions presented in this study are included in the article. Further inquiries can be directed to the corresponding author.

## Abbreviations

The following abbreviations are used in this manuscript:

CON	(negative) control
DM	Dry matter
LAB	Lactic acid bacteria
LABhe	Heterofermentative lactic acid bacteria
LABho	Homofermentative lactic acid bacteria
